# Longitudinal within-host evolution of HIV Nef-mediated CD4, HLA and SERINC5 downregulation activity: a case study

**DOI:** 10.1186/s12977-019-0510-1

**Published:** 2020-01-09

**Authors:** Hanwei Sudderuddin, Natalie N. Kinloch, Steven W. Jin, Rachel L. Miller, Bradley R. Jones, Chanson J. Brumme, Jeffrey B. Joy, Mark A. Brockman, Zabrina L. Brumme

**Affiliations:** 1 Faculty of Health Sciences, Simon Fraser University, Burnaby, BC V5A 1S6 Canada; 2 BC Centre for Excellence in HIV/AIDS, Vancouver, BC Canada; 3 Department of Medicine, University of British Columbia, Vancouver, BC Canada

**Keywords:** Nef, HIV evolution, Longitudinal, CD4, HLA, SERINC5

## Abstract

The HIV accessory protein Nef downregulates the viral entry receptor CD4, the Human Leukocyte Antigen (HLA)-A and -B molecules, the Serine incorporator 5 (SERINC5) protein and other molecules from the infected cell surface, thereby promoting viral infectivity, replication and immune evasion. The *nef* locus also represents one of the most genetically variable regions in the HIV genome, and *nef* sequences undergo substantial evolution within a single individual over the course of infection. Few studies however have simultaneously characterized the impact of within-host *nef* sequence evolution on Nef protein function over prolonged timescales. Here, we isolated 50 unique Nef clones by single-genome amplification over an 11-year period from the plasma of an individual who was largely naïve to antiretroviral treatment during this time. Together, these clones harbored nonsynonymous substitutions at 13% of *nef’*s codons. We assessed their ability to downregulate cell-surface CD4, HLA and SERINC5 and observed that all three Nef functions declined modestly over time, where the reductions in CD4 and HLA downregulation (an average of 0.6% and 2.0% per year, respectively) achieved statistical significance. The results from this case study support all three Nef activities as being important to maintain throughout untreated HIV infection, but nevertheless suggest that, despite *nef’*s mutational plasticity, within-host viral evolution can compromise Nef function, albeit modestly, over prolonged periods.

## Background

HIV is an enveloped retrovirus with extensive capacity for mutation and within-host genetic diversification [1–4], which occur as a result of reverse transcriptase errors [5], viral recombination [6] and sublethal APOBEC3G-mediated mutagenesis [7] combined with a short viral generation time and high viremia during untreated infection [4]. In most cases of HIV transmission, a single transmitted/founder virus initiates productive infection in the new host [8–10], but descendant within-host HIV populations rapidly diversify and undergo successive genetic bottlenecks under selection pressures by host antiviral immune responses [11–15].

Of all the HIV genes, *nef* displays particularly high rates of within-host viral diversification and evolution [16–18]. Nef is also a determinant of HIV pathogenesis [19], and performs various functions that promote viral infectivity, replication and immune evasion [19–21]. Nef’s ability to downregulate CD4 and Human Leukocyte Antigen (HLA)-A and -B molecules from the infected cell surface represent two of its most widely studied functions [22–24]. Nef-mediated CD4 downregulation prevents cellular superinfection [25], allows infected cells to evade antibody-dependent cell-mediated cytotoxicity (ADCC) responses by abrogating CD4-induced Env conformational changes required for antibody binding [26, 27], and enhances Env incorporation into budding virions [28]. Nef-mediated HLA-A and -B downregulation allows HIV-infected cells to evade HLA-restricted CD8+ cytotoxic T lymphocyte (CTL) responses [29, 30]. More recently, Nef has been found to internalize the transmembrane host restriction factor Serine incorporator 5 (SERINC5), thereby preventing its inclusion into budding HIV virions and enhancing viral infectivity [31, 32]. We and others have observed that all three of these functions are attenuated in Nef clones isolated from HIV elite controllers who spontaneously suppress plasma viremia to < 50 RNA copies/mL in the absence of therapy [33–37], suggesting that variation in Nef activity contributes to biologic outcomes.

Though *nef* undergoes substantial within-host evolution [38–41], studies characterizing the relationships between primary *nef* sequences and the functions of their corresponding expressed proteins have predominantly been cross-sectional, with one or a few *nef* sequences evaluated per participant at a single timepoint [34, 42–47]. Few studies have simultaneously assessed within-host genetic [48, 49] and functional Nef evolution over long timescales [50, 51], and none to our knowledge have investigated Nef-mediated SERINC5 downregulation longitudinally. The impact of long-term within-host *nef* evolution on Nef protein function thus remains unclear. To address this, we isolated 50 unique Nef clones by single-genome amplification over an 11-year period in an individual who was largely naïve to antiretroviral treatment, and assessed their ability to downregulate CD4, HLA and SERINC5 molecules.

## Results

**Fig. 1:**
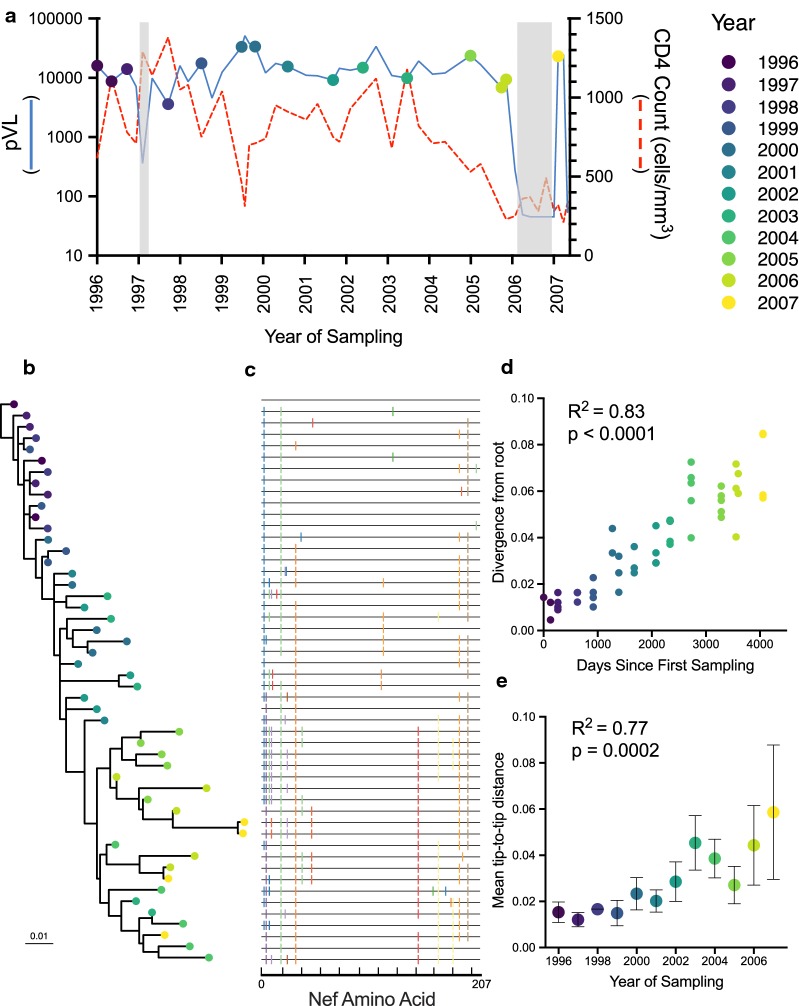
Evolution of within-host *nef* sequences. **a** Participant plasma viral load (solid blue line), CD4+ T-cell count (red dotted line) and sampling history (colored circles). Grey shading indicates periods on cART. **b** Maximum likelihood phylogenetic tree inferred from 50 unique within-host *nef* sequences, where the root represents the inferred most recent common ancestor (MRCA). Scale in estimated substitutions per nucleotide site. **c** Within-host Nef amino acid alignment, with sequences ordered according to the phylogeny, where the top sequence denotes the master and colored lines in the below sequences represent nonsynonymous substitutions with respect to it. Tickmarks on the X-axis are placed every 20 amino acids. **d** Linear relationship relating root-to-tip phylogenetic distances to sampling time; this analysis quantifies within-host HIV sequence *divergence* from the root over time. **e** Linear relationship relating average tip-to-tip phylogenetic distances between clonal sequences sampled each year to sampling time; this analysis quantifies within-host HIV sequence *diversity* over time. Colored dots represent the mean tip-to-tip phylogenetic distance and error bars show standard deviation

The study participant, a male, was diagnosed with HIV in August 1996. Over the following 11 years, he did not receive antiretroviral treatment except for a short period between August and November 1997, and again from August 2006 to July 2007 (Fig. 1a). From study entry in 1996 until August 2006, the participant’s plasma viral load remained relatively stable at a median of 4.1 log_10_ copies HIV RNA/mL, while his CD4+ T cell count declined by an average of 50 cells/mm^3^ per year (R^2^ = 0.28, p < 0.0001), reaching a nadir of 230 cells/mm^3^ in June 2006. A total of 113 plasma HIV RNA *nef* sequences, sampled at 15 timepoints between August 1996 and September 2007, were previously isolated from the participant by single-genome amplification [40]. From this original dataset, we selected a minimum of 3 *nef* sequences per year, totaling 50 unique sequences, to represent within-host *nef* genetic diversity and evolution over the study period (Fig. 1b and Additional file 1). These *nef* sequences differed from one another at 97 of 621 (15.6%) nucleotides and 27 of 207 (13%) amino acids (Fig. 1c). The selected sequences captured all major within-host selective sweeps and represented 70.4% of the amino acid diversity within the original dataset of 113 *nef* sequences (of these, 71 were unique at the amino acid level; we selected 50 for study, yielding 70.4% coverage) [40].

Longitudinally-sampled gene sequences can be used to infer molecular phylogenies on natural timescales and to estimate the location and timing of the tree root, representing the most recent common ancestor, or MRCA, of the dataset [52]. Indeed, analysis of the original plasma HIV RNA sequence dataset using Bayesian approaches yielded a root date estimate of December 1995 [40], consistent with the participant having been infected in the year before diagnosis. For the present analysis, we reconstructed *nef*’s within-host evolution by inferring a maximum-likelihood phylogeny from an alignment of the 50 selected *nef* sequences, and identified the ‘best fit’ root position using the software package TEMPoral Exploration of Sequences and Trees (TempEst) (Fig. 1b) [52]. We observed a significant linear relationship between root-to-tip distance, a measure of within-host HIV divergence from the MRCA, and sampling date (R^2^ = 0.83, p < 0.0001; Fig. 1d). We also observed a significant linear relationship between the average patristic (phylogenetic tip-to-tip) distance between all clones isolated in a given year, a measure of within-host diversity, and sampling date (R^2^ = 0.77, p = 0.0002; Fig. 1e). These observations indicate strong molecular clock signal in the data, and are consistent with increases in root-to-tip divergence and population viral diversity that typify within-host HIV evolution [53, 54].

**Fig. 2:**
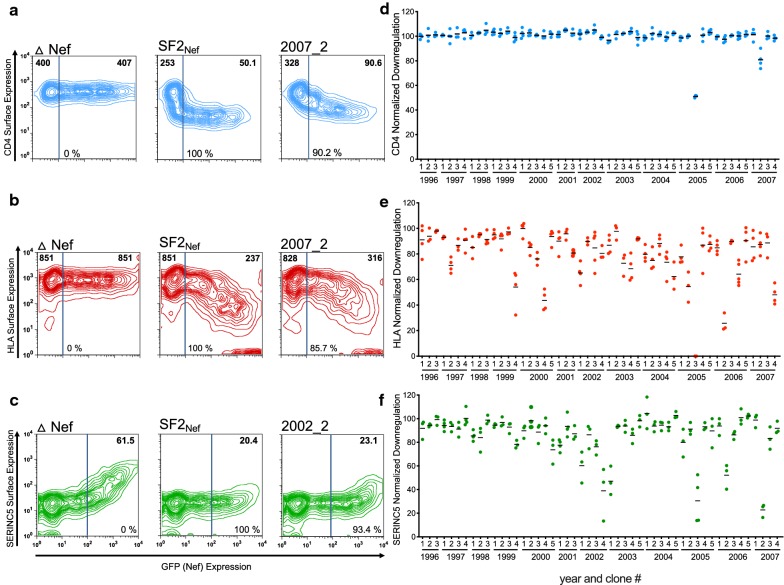
Function of within-host *nef* clones. **a**–**c** Representative flow cytometry plots showing CD4 (blue), HLA (red) and SERINC5 (green) downregulation activities of select participant-derived Nef clones and controls. The numbers in bold within each plot denote the median fluorescence intensity (MFI) of receptor expression in that gate. The number at the bottom of each plot denotes each Nef clone’s function normalized to that of the positive control SF2_NEF_. **d** SF2_NEF_-normalized CD4 downregulation activity of the 50 Nef clones. Each clone was independently assayed a minimum of 3 times; a total of 189 replicates are represented here. **e** SF2_NEF_-normalized HLA downregulation activity of the 50 Nef clones (183 total replicates). **f** SF2_NEF_-normalized SERINC5 downregulation activity of the 50 Nef clones (157 total replicates). Horizontal bars show mean normalized function for each clone

**Fig. 3:**
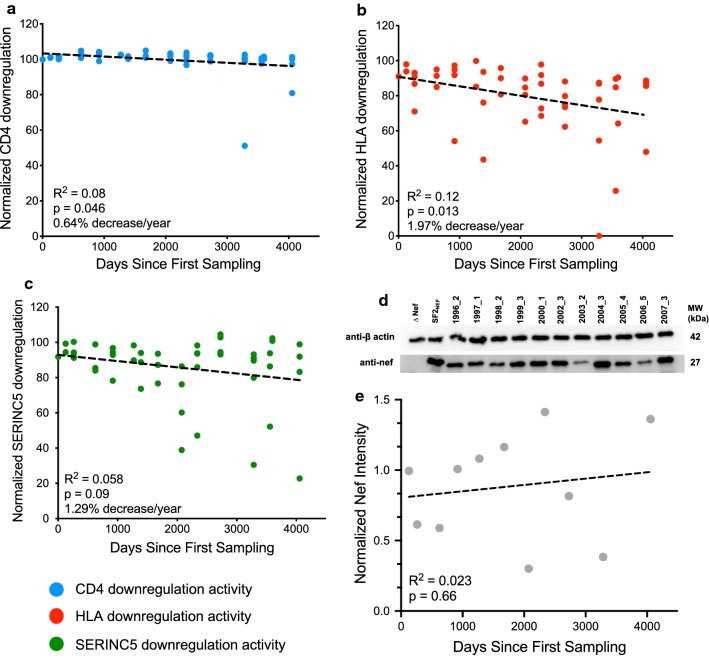
Nef functional evolution over time. Nef-mediated CD4 downregulation (**a**), HLA downregulation (**b**) and SERINC5 downregulation (**c**) over time. **d** Western blot of each year’s maximally functioning Nef clone along with cellular β-actin level. Empty pSELECT-GFP (∆Nef) and pSELECT-GFP vector with SF2_NEF_ served as negative and positive controls respectively. **e** Nef western blot intensities, normalized to that of cellular β-actin levels, over time

Each *nef* sequence was cloned into a reporter plasmid that also expressed the green fluorescent protein (GFP) from a separate promoter [46], and was assessed for its ability to downregulate cell surface CD4, HLA-A*02 (as a representative HLA class I allele), and SERINC5 in an immortalized CEM-derived CD4+ T-cell line by flow cytometry as described in [33, 44, 46] (Fig. 2a–c). The function of each Nef clone was normalized to that of the HIV subtype B SF2 reference strain (SF2_NEF_), such that normalized values above or below 100% represented downregulation functions that were higher or lower than SF2_NEF_, respectively. Each Nef clone was assayed a minimum of three times in independent experiments (Fig. 2d–f). All Nef clones exhibited some level of function in all three assays, with the exception of clone 2005_3, which was completely defective for HLA downregulation and ranked in the bottom 5th percentile for CD4 and SERINC5 downregulation (51.1% and 30.5% activity, respectively). Replicate measurements of each Nef clone were highly consistent (Fig. 2d–f): standard deviations between replicate measurements were, on average, 2.3% for CD4 downregulation, 6.2% for HLA downregulation and 5.9% for SERINC5 downregulation. Each clone’s function was subsequently reported as the mean of all replicate measurements (Fig. 3a–c).

Altogether, the 50 within-host Nef clones displayed a relatively narrow range of CD4 downregulation function (median 101% [Q1–Q3 = 99–103%] relative to SF2_NEF_; Fig. 3a), whereas wider ranges were observed for HLA downregulation function (median 86% [Q1–Q3 = 74–93%], Fig. 3b) and SERINC5 downregulation function (median 92% [Q1–Q3 = 82–97%], Fig. 3c). Linear models relating each clone’s function to its sampling date revealed that, on average, Nef-mediated CD4 downregulation function declined by 0.64% per year (R^2^ = 0.08; p = 0.046, Fig. 3a) while HLA downregulation function declined on average by 1.97% per year (R^2^ = 0.12; p = 0.013, Fig. 3b). Nef-mediated SERINC5 downregulation function also declined on average by 1.29% per year, but this did not achieve statistical significance (R^2^ = 0.058; p = 0.09, Fig. 3c). Consistent with the results of the temporal analyses, we also observed strong negative relationships between Nef function and divergence from the root (CD4 downregulation R^2^ = 0.092, p = 0.032; HLA downregulation R^2^ = 0.13, p = 0.010 and SERINC5 downregulation R^2^ = 0.066, p = 0.072; data not shown), corroborating the notion that accumulating substitutions gradually compromise Nef function. Recognizing that defective HIV sequences can naturally arise in plasma but could confound our results, we confirmed that the temporal decreases in Nef function remained after exclusion of 2005_3, the clone that was defective for HLA downregulation and highly attenuated for the other functions. The results of this sensitivity analysis were consistent with the original findings (CD4 downregulation: R^2^ = 0.13, p = 0.01; HLA downregulation: R^2^ = 0.11, p = 0.02; and SERINC5 downregulation: R^2^ = 0.04, p = 0.16; data not shown).

While the major genetic determinants of Nef function are genetically separable [55–57], previous studies of natural *nef* sequences have demonstrated modest correlations between certain Nef activities [33, 35, 46, 47], suggesting the presence of secondary or shared genetic determinants. Consistent with this, Nef-mediated CD4 and HLA downregulation functions of the 50 studied clones correlated weakly (Spearman R = 0.35; p = 0.013), as did Nef-mediated CD4 and SERINC5 downregulation functions (Spearman R = 0.24; p = 0.047) (data not shown). No correlation however was observed between HLA and SERINC5 downregulation functions (Spearman R = 0.17, p = 0.12, data not shown).

We also undertook exploratory analyses to identify residues most associated with Nef functional reductions in our dataset, noting of course that, since all sequences descend from a common ancestor and substitutions accumulate in the population over time, identified residues correlate with, but do not necessarily individually cause, reduced function. The results are presented in Additional file 2. Consistent with some shared genetic determinants of Nef function, the two residues most strongly associated with reductions in CD4 downregulation were E149D and P25X (p < 0.01; q < 0.1), those most associated with reductions in HLA downregulation were V33A and S8X (p < 0.01; q < 0.1) while those most associated with reductions in SERINC5 downregulation were P25X and M168I (p < 0.01; q < 0.2). Though the extremely strong linkage between some codons precluded us from performing a reliable multivariable analysis, our dataset did include two natural examples where a single substitution likely abrogated one or more Nef functions. Clone 2005_3 for example, which was completely defective for HLA downregulation and ranked in the bottom 5th percentile of clones in terms of CD4 and SERINC5 downregulation, differed from clone 2005_4, which was functional for all three activities, by only the W141L substitution. Similarly, clone 2007_2, which ranked in the bottom 10th percentile for both CD4 and SERINC5 downregulation, differed from clone 2007_3, which was functional for all three activities, by only the G41E substitution. No other sequences in the dataset exhibited W141L or G41E, which are exceedingly rare or nonexistent in natural isolates (the Los Alamos HIV database reports their frequencies as 0% and 0.44%, respectively, in HIV subtype B; https://www.hiv.lanl.gov). Together this suggests that these mutations are responsible for the dramatic yet highly specific functional defects of these clones.

Finally, we investigated whether steady-state Nef expression, measured by Western Blot for each year’s maximally functioning clone (defined in terms of normalized Nef-mediated CD4 and HLA downregulation), changed appreciably over time (Fig. 3d). After normalization to cellular β-actin levels however, no consistent alterations in steady-state Nef expression were observed over the study period (R^2^ = 0.023; p = 0.66, Fig. 3e).

## Conclusions

Our study of within-host HIV *nef* function over an 11-year period revealed a number of insights. First, it confirmed marked within-host evolution in *nef* (the 50 studied clones differed from one another at 13% of Nef’s codons, a value that is comparable to previous reports, e.g. [48]). Secondly, it revealed that Nef’s individual activities differed widely in terms of their dynamic ranges of function. Nef-mediated CD4 downregulation was particularly conserved: all but two clones exhibited CD4 downregulation functions of > 81% (relative to SF2_Nef_), and the 25th to 75th percentile of clones exhibited CD4 downregulation functions between 99 and 103%. In contrast, Nef-mediated HLA downregulation ranged from 0 to 99.8% between clones while SERINC5 downregulation ranged from 22.8 to 104.5%. Strong conservation of Nef-mediated CD4 downregulation, yet wider ranges for other functions mirrors observations from cross-sectional, population-based studies [37, 44, 46] and suggests that the latter type of study may benefit from isolating multiple Nef clones per participant for better representation. Thirdly, despite substantial within-host *nef* evolution, Nef-mediated CD4, HLA and SERINC5 downregulation functions and steady-state Nef protein expression levels were, on the whole, remarkably conserved, suggesting that a certain amount of selective pressure to preserve these Nef properties is maintained throughout infection [58, 59].

Our fourth key observation was that, despite overall maintenance of all three Nef functions, all nevertheless declined modestly over time. CD4 downregulation function declined most slowly (0.64% per year), whereas HLA downregulation and SERINC5 function declined somewhat more rapidly (1.97% and 1.29% per year, respectively, though the latter did not reach statistical significance). For reference, CD4 T-cell counts declined by 5% per year on average (assuming a baseline CD4 count of 1000 cells/mm^3^). The observation that CD4 downregulation displays the narrowest functional range and the slowest temporal decline suggests that, of the three functions, it is the most critical to maintain in vivo. In contrast, the broader functional ranges and the faster rates of decline observed for HLA and SERINC5 downregulation suggest that selective pressure to preserve these functions may wane to some extent during advanced infection, perhaps because other viral mutations emerge that reduce the importance of this function. Indeed, while the observed overall ~ 20% reduction in Nef-mediated HLA downregulation over the study period would likely compromise immune-mediated recognition of infected cells (evidence to support this comes from experiments demonstrating that the extent of Nef-mediated HLA downregulation on target cells inversely correlates with the ability of peptide/HLA-specific effector T cells to recognize them in vitro [60, 61]), the selection and accumulation of immune escape mutations across the HIV genome gradually erodes the importance of this function [62, 63]. In fact, mutational immune escape is readily apparent in the data [40]: analysis of the participant’s earliest *nef* sequences using the epitope-prediction software NetMHCpan4.0 [64] in context of their HLA class I profile (determined to be A*26:01/A*30:01, B*13:02/B*14:01, C*06:02/C*08:02) revealed a predicted HLA-B*13-restricted epitope spanning Nef codons 124–133, W**N**NYTPGPGV, present in all Nef sequences originally isolated at the baseline (August 1996) timepoint [40]. This sequence rapidly escaped to the B*13-adapted form W**H**NYTPGPGV and subsequently became fixed in the population, providing one (of surely many) examples of immune escape mutations across the viral genome that would, over time, likely reduce the importance of Nef’s continued ability to downregulate HLA-A and HLA-B molecules.

The major caveat of this study is that, since only a single individual was studied, the results may not be broadly generalizable. Furthermore, though we functionally characterized 50 unique Nef sequences that displayed strong molecular clock signal and other characteristic properties of within-host HIV evolution, these do not capture all within-host variants that would have emerged during the study period. Larger within-host Nef genotype/phenotype studies, including those that additionally assess proviral Nef sequences persisting during long-term cART, will shed further light on the extent to which Nef’s immune evasion and infectivity enhancing functions evolve during untreated HIV infection and are preserved in the HIV reservoir. Despite these limitations, our case study nevertheless reveals that, for all of *nef’s* mutational plasticity, within-host viral evolution can gradually erode its protein function—albeit modestly—over prolonged timescales.

## Methods

### HIV RNA extraction and single-genome amplification of Nef

An individual living with HIV, for whom blood plasma had been longitudinally sampled at 15 timepoints over an 11-year period, was studied (Fig. 1a) [40]. The participant provided written informed consent and this study was approved by the Providence Health Care/University of British Columbia and Simon Fraser University research ethics boards.

As described in [40], HIV RNA was extracted from plasma using the BioMerieux NucliSENS EasyMag system and *nef* was amplified using limiting-dilution nested RT-PCR such that no more than 25–30% of resulting reactions would be positive. Amplicons were sequenced on an ABI 3130xl automated DNA analyzer, and chromatograms were edited in Sequencher version 5.0 software (GeneCodes). After excluding *nef* sequences that contained nucleotide mixtures, hypermutations (identified using HyperMut v2.0 [65]) or other defects, a total of 113 intact plasma HIV *nef* sequences remained. From these, 50 unique *nef* sequences were selected to maximize temporal and HIV genetic coverage of the dataset. Genbank Accession numbers of the 50 sequences are: MG822918, MG822920, MG822921, MG822925, MG822927–MG822929, MG822934, MG822935, MG822937, MG822938, MG822941, MG822943–MG822946, MG822950–MG822955, MG822957, MG822959, MG822960, MG822963, MG822964, MG822968, MG822971, MG822974, MG822977, MG822979, MG822982, MG822983, MG822987, MG822988, MG822991, MG822993–MG822995, MG822998–MG823001, MG823003–MG823005, MG823007, MG823011 and MG823014.

The original first-round RT-PCR amplicons (generated using High-Fidelity enzymes as described in [40]) were used as templates to generate new second-round amplicons using primers containing restriction enzyme sites, as follows. The forward primer was 5′-AGAGCACC**GGCGCGCC***TCCACATACCTASAAGAATMAGACARG*-3′ (the AscI site is in bold; italics denote the HIV-specific sequence spanning HXB2 nucleotides 8746 to 8772) and the reverse primer was 5′-GCCT**CCGCGG**ATCGAT*CAGGCCACRCCTCCCTGGAAASKCCC*-3′ (the SacII site is in bold; HXB2 nucleotides 9474 to 9449 are italicized). A high-fidelity polymerase was used (Roche Expand Hifi System). Amplicons were run on a 1% agarose gel, excised and purified (Thermoscientific^®^ GeneJET Gel Extraction Kit).

*Nef* amplicons were cloned into a modified pSELECT-GFPzeo vector containing AscI and SacII restriction sites within its multiple cloning site [47]. As described in [47], *nef* amplicons were digested with AscI and SacII, ligated into cut pSELECT-GFPzeo (T4 ligase; Thermo Fisher^®^), and transformed into chemically competent *E. coli* (E. cloni 10G DUOs; Lucigen). Plasmid DNA from a minimum of three colonies per transformation was isolated, purified (Thermo Fisher^®^ OMEGA EZNA plasmid minikit) and re-sequenced to confirm identity. All 50 *nef* sequences were identical at the amino acid level to the sequence originally generated by single-genome amplification: 36 (72.0%) were also identical at the nucleotide level, while 14 harbored a single nucleotide difference that encoded a synonymous substitution.

Within-host *nef* sequences were aligned using HIV Align (options: MAFFT v7 [66]; codon alignment). Maximum likelihood phylogenetic inference was performed using PhyML v3.0 [67] under a general time-reversible (GTR) substitution model. The tree was rooted using TempEST v1.5.1, which identifies the root location that minimizes the sum of the squared residuals from a regression line relating the root-to-tip phylogenetic distances and collection dates of the sequences in the dataset, where this root position represents an estimate of the timing of the MRCA of the dataset [52]. The amino acid “highlighter” plot was generated in R using the ggtree package [68].

### Nef-mediated CD4, HLA and SERINC5 downregulation assays

Each Nef clone was assayed in at least three independent experiments for its CD4, HLA and SERINC5 downregulation capacity using assays as described in [33, 44, 46]. Briefly, CD4 and HLA downregulation functions were assessed by transfecting *nef* plasmid DNA into a CEM-derived CD4+ T-cell line engineered to stably express HLA-A*02 (CEM-A*02) [46]. The *nef* allele from the HIV subtype B reference strain SF2 (SF2_NEF_) served as a positive control and empty pSELECT-GFPzeo (∆Nef) served as a negative control. For each participant-derived or control *nef* sequence, 4 μg of *nef* plasmid DNA was delivered into 500,000 CEM-A*02 cells via electroporation (BioRad GenePulser MXCell™ instrument) in 96-well plates. One positive and one negative control plasmid were included for every 6 study samples in each experiment. Cells were incubated for 20 to 24 h, and then stained with allophycocyanin-labeled anti-CD4 and phycoerythrin-labeled anti-HLA-A*02 antibodies (BD Biosciences). Cell surface expression of CD4 and HLA were measured using flow cytometry (Millipore Guava 8HT). The CD4 and HLA downregulation functions of participant-derived Nef clones were normalized to those of the positive control, SF2_NEF_, using the following equation [47]:

$$\left\{ { 1 { }{-} \, \left[ {{\text{MFI}}_{\text{clone}} \left( {{\text{GFP}}^{ + } } \right)/{\text{MFI}}_{\text{clone}} \left( {{\text{GFP}}^{ - } } \right)} \right]} \right\}/\left\{ { 1 { }{-} \, \left[ {{\text{MFI}}_{\text{SF2}} \left( {{\text{GFP}}^{ + } } \right)/{\text{MFI}}_{\text{SF2}} \left( {{\text{GFP}}^{ - } } \right)} \right]} \right\},$$

where MFI is median fluorescence intensity within the indicated GFP gate (a surrogate of Nef expression).

To assess Nef-mediated internalization of SERINC5 from the cell surface, 1 × 10^6^ CEM-A*02 T cells were co-transfected with 1 μg of pSELECT-GFPzeo encoding *nef* and 5 μg of pSELECT-SERINC5-internal HA tag (iHA)-∆GFP by electroporation in 150 μL OPTI-mem medium (Thermo Fisher), as described in [37]. The pSELECT-SERINC5-iHA-∆GFP was sub-cloned from the pBJ5-SERINC5(iHA) described in [32]. Cultures were incubated for 20 to 24 h and subsequently stained with 0.5 μg of Alexa Fluor^®^ 647 anti-HA.11 (BioLegend) and analyzed by flow cytometry. Nef-mediated SERINC5 downregulation was normalized to the positive and negative controls using the following formula:

$$\left( {{\text{MFI}}_{{\Delta {\text{Nef}}}} - {\text{MFI}}_{\text{clone}} } \right)/\left( {{\text{MFI}}_{{\Delta {\text{Nef}}}} - {\text{MFI}}_{\text{SF2}} } \right) \times 100,$$

where stated MFI measurements correspond to those in the GFP + (Nef-expressing) gate.

### HLA class I typing

Human Leukocyte Antigen (HLA) class I typing was performed by locus-specific nested PCR followed by bulk DNA sequencing as described in [69].

### Western blotting

Western blotting was performed for the maximally functioning clones from years 1996–2000 and 2002–2007. A total of 2.5 × 10^6^ CEM cells were transfected with 10 μg of participant-derived or control (SF2_NEF_) plasmid DNA, and cell pellets were harvested following 24 h of incubation. Cells were lysed with Nonidet P-40 lysis buffer (1% Nonidet P-40, 50 mM Tris HCl, 150 mM NaCl) containing a protease inhibitor cocktail (P8340; Sigma). The lysed cells were centrifuged and the resultant supernatants were subjected to SDS-PAGE, with the protein electroblotted onto a PVDF membrane. Nef was detected using sheep polyclonal anti-HIV Nef serum (1:2000 dilution; NIH AIDS Research and Reference Reagent Program, USA) primary antibody, followed by horseradish peroxidase (HRP)-conjugated donkey anti-sheep IgG (1:35,000; GE Healthcare). Blots were visualized using an ImageQuant LAS 4000 chemiluminescent imager (GE Healthcare). Nef intensity was quantified using ImageJ analysis software, and was performed by normalizing the intensity of each Nef band to its corresponding β-actin control [70].

### Statistical analysis

Statistical analyses were performed in PRISM v.8.0.2 (Graphpad). The phylogenetic tree was visualized using the ape package (v5.3) and the ggtree package in R [68, 71]. Patristic distances were extracted from the maximum likelihood phylogeny using the cophenetic.phylo function from the ape package (v5.3) in R [71]. Root-to-tip distances were extracted from the maximum likelihood phylogeny using the node.depth.edgelength function from ape package (v5.3) in R [71]. The Mann–Whitney U-test was used to test for relationships between every amino acid observed at every position in the within-host *nef* alignment and each of the three Nef functions. Here, multiple comparisons were addressed using q-values, the p-value analogue of the false discovery rate, defined as the expected proportion of false positives among results deemed significant at a given p-value threshold (e.g. at a q ≤ 0.2, we expect 20% of identified associations to be false positives) [72].

## Supplementary information

**Additional file 1:** Nef sequences and related functional data.

**Additional file 2:** Codon-by-codon analysis of Nef sequence with function.

## Data Availability

The *nef* sequence dataset supporting the conclusions of this article are available in the Genbank repository (see Accession numbers above). The dataset (Nef amino acid sequences linked to functional measurements) supporting the conclusions of this article is included as Additional file 1. The codon-by-codon analyses of Nef sequence with function are contained within Additional file 2.

## Abbreviations

ADCC: antibody-dependent cell-mediated cytotoxicity
cART: combined antiretroviral therapy
CTL: cytotoxic T-lymphocyte
HLA: Human Leukocyte Antigen
MRCA: most recent common ancestor
MFI: median fluorescence intensity
PCR: polymerase chain reaction
SERINC5: Serine incorporator 5
